# Editorial: Cancer risk in patients with acromegaly – is extensive screening needed?, volume II

**DOI:** 10.3389/fendo.2026.2004968

**Published:** 2026-10-09

**Authors:** Jakob Dal, Raluca Alexandra Trifanescu

**Affiliations:** 1 Department of Endocrinology, Aalborg University Hospital, Aalborg, Denmark; 2 Steno Diabetes Center North Jutland, Aalborg, Denmark; 3 Carol Davila University of Medicine and Pharmacy, Bucharest, Romania

**Keywords:** acromegaly, cancer, growth hormone, IGF1, pituitary adenoma

Acromegaly is a rare endocrine disorder characterised by chronic hypersecretion of growth hormone (GH) and insulin-like growth factor 1 (IGF-1). It is associated with multiple comorbidities, including hypertension, diabetes mellitus, musculoskeletal complications, sleep apnoea, colonic polyps, and neoplasia, which adversely affect quality of life and mortality (1, 2).

Diagnosis of acromegaly is frequently delayed by 4–10 years between symptom onset and diagnosis (2–4). Diagnostic delay appears particularly pronounced among older patients and women and has been associated with larger pituitary tumours, increased cavernous sinus invasion, and a lower likelihood of biochemical remission (5–7). Prolonged exposure to GH and IGF-1, resulting from delayed diagnosis or persistent disease activity, may contribute to development of neoplasia. Both hormones have mitogenic and anti-apoptotic effects and may promote angiogenesis and a tumour-permissive microenvironment. However, the epidemiological association between acromegaly and cancer remains controversial, and the extent to which persistent hormonal excess independently contributes to cancer development and progression remains incompletely understood (8).

The current Frontiers in Endocrinology Research Topic, “Cancer risk in patients with acromegaly – is extensive screening needed?”, provides several interesting new perspectives on this question. The collection addresses breast and colorectal cancer and, importantly, also expands our understanding of tumour biology through studies of somatic *GNAS* mutations, McCune–Albright syndrome (MAS), and the gut microbiome.

Breast cancer is one of the most common malignancies in women, but whether acromegaly confers an additional risk remains uncertain. Lee et al. performed a systematic review of 24 studies published between 1957 and 2025, including 17,413 patients with acromegaly. Reported breast cancer prevalence ranged from 0.42% to 5.85%, with some variation according to study design. However, these differences were not statistically significant, and the pooled standardised incidence ratio (SIR) from 11 studies was 1.20 (95% CI 0.94–1.54).

Thus, there was no clear evidence of an increased breast cancer risk. Interestingly, registry-based studies generally suggested neutral or only modestly increased risk, whereas some single-centre and prospective studies reported higher incidence. Studies examining GH and IGF-1 concentrations have likewise produced conflicting results, with some reporting lower IGF-1 levels among patients with breast cancer and others finding no differences (8). These discrepancies may reflect surveillance bias, differences in study design, and limited adjustment for confounding factors. Overall, the findings are reassuring and support breast cancer screening according to recommendations for the general population rather than intensified surveillance solely on the basis of acromegaly (Lee et al.).

The picture is different for colorectal neoplasia. Several previous studies have suggested an increased risk of colorectal cancer in acromegaly (9–12). A recent nationwide registry study with 44 years of follow-up further supports this association, reporting an increased risk of colorectal cancer (IRR 1.78, 95% CI 1.22–2.60), while overall cancer risk was not significantly increased. Notably, colorectal cancers were more often localized then disseminated, suggesting that increased detection may contribute to the observed association (13).

These findings provide an important background for the contribution by Grifoni et al. who explore a potential biological link between acromegaly and colorectal carcinogenesis.

Grifoni et al. discuss alterations in the gut microbiota in patients with acromegaly and their potential relevance to colorectal cancer risk. Previous studies have described reduced faecal microbial diversity and changes in the relative abundance of several bacterial taxa, including a predominance of Bacteroidetes and a reduced Firmicutes/Bacteroidetes ratio, together with lower representation of Bifidobacterium, Oscillospira, and Dialister (14). Grifoni and colleagues propose that gut dysbiosis may interact with GH/IGF-1 excess, insulin resistance, and inflammatory signalling to create a tumour-promoting environment. Although this hypothesis remains to be demonstrated, it is an intriguing concept that moves the discussion beyond the traditional focus on GH and IGF-1 and raises the possibility that the gut microbiome may represent an additional pathway linking acromegaly to colorectal neoplasia.

The Research Topic also provides important insights into the molecular biology of acromegaly. Dillon et al. investigated the clinical relevance of somatic *GNAS* mutations by combining a meta-analysis with a series of 22 patients who underwent pituitary tumour resection between 2022 and 2024. Somatic *GNAS* mutations were identified in 41% of tumours, consistent with their meta-analysis of 55 studies reporting a prevalence of approximately 38%. *GNAS*-positive tumours were smaller and appeared less invasive at diagnosis. In the surgical series, patients with *GNAS*-positive tumours were older at surgery, and most tumours were classified as mammosomatotroph adenomas. They also demonstrated greater GH suppression following acute octreotide administration. However, most previous studies have not demonstrated a consistent association between *GNAS* status and biochemical response to somatostatin analogues. Thus, *GNAS* mutations appear to provide valuable biological insight, but their current role as prognostic or predictive biomarkers remains uncertain.

Boguslawska et al. further illustrate the clinical relevance of *GNAS*-related disease through a case of McCune–Albright syndrome (MAS) presenting with acromegaly, severe kyphoscoliosis, tertiary hyperparathyroidism, subclinical hyperthyroidism, and café-au-lait spots. Genetic testing identified a *GNAS* splice-site variant of uncertain significance, while 18F-FDG PET/CT showed no neoplastic lesions apart from the pituitary adenoma. The case highlights that the combination of acromegaly with skeletal deformities, café-au-lait pigmentation, and thyroid or parathyroid abnormalities should prompt consideration of MAS. Although genetic confirmation may be challenging, recognition of the clinical phenotype remains important.

Finally, data from a large Chinese cohort of 1,692 patients (Bai et al.) emphasise that diagnostic delay remains clinically relevant. Older age at diagnosis and longer diagnostic delay were associated with a greater burden of comorbidities. Interestingly, the diagnostic delay was 4.4 years, suggesting improving clinical recognition (Bai et al.) Among patients diagnosed after age 50 years, hypertension and hyperlipidaemia were more common in women than men, highlighting the emerging importance of sex differences in the clinical manifestations of acromegaly (15, 16).

Taken together, the contributions to this Research Topic reinforce the complexity of cancer risk in acromegaly. Current evidence does not support enhanced breast cancer screening, whereas the association with colorectal neoplasia appears more consistent and may involve mechanisms extending beyond GH and IGF-1, including alterations in the gut microbiome. At the same time, *GNAS* mutations and MAS illustrate the molecular and clinical heterogeneity of acromegaly. Overall, our understanding of cancer risk in acromegaly has taken an important step forward.
